# Effect of Copper Alloying on Hydrogen Embrittlement of Fe-28Mn-10Al-1C Austenitic Low-Density Steel

**DOI:** 10.3390/ma18174139

**Published:** 2025-09-04

**Authors:** Jiahao Gu, Sifan Jiang, Yanfei Qi, Xiqiang Ren, Yungang Li

**Affiliations:** 1 College of Metallurgy and Energy, North China University of Science and Technology, Tangshan 063210, China; 15517164063@163.com (J.G.); lyg@ncst.edu.cn (Y.L.); 2 Faculty of Chemical Engineering and Energy Technology, Shanghai Institute of Technology, Shanghai 201418, China; 15239620588@163.com; 3 College of Mechanical Engineering, Tangshan Polytechnic University, Tangshan 063299, China; rxq0219@126.com

**Keywords:** Fe-Mn-Al-C low-density steel, copper alloying, hydrogen trapping, hydrogen embrittlement susceptibility

## Abstract

This study investigated the hydrogen embrittlement behavior of Fe-28Mn-10Al-1C-(0,3) Cu austenitic low-density steels after hydrogen charging. Electrochemical hydrogen charging and thermal desorption spectroscopy (TDS) were employed to characterize hydrogen desorption behavior and identify hydrogen trap types in cold-rolled (LZ) and annealed (TH) conditions. Uniaxial tensile tests were conducted to obtain mechanical properties and the hydrogen embrittlement index (*HEI*), enabling quantitative evaluation of hydrogen embrittlement susceptibility. Fracture surface morphology was analyzed to elucidate the underlying embrittlement mechanisms. Results indicate that annealing treatment and Cu addition have negligible effects on the activation energy of reversible hydrogen traps, suggesting similar trap types. The reversible hydrogen content decreased by 0.1 wt.ppm and 0.2 wt.ppm in LZ-3Cu and TH-3Cu, respectively, compared to their Cu-free counterparts, indicating that Cu addition mitigates the accumulation of reversible hydrogen. Annealed specimens exhibited lower *HEI* values, with the *HEI* of TH-0Cu decreasing from 21.3% to 13.5% and that of TH-3Cu reaching only 9.6%. Fracture mode transitioned from mixed brittle-ductile to fully ductile with Cu alloying, accompanied by a shift from the coupled the Hydrogen-Enhanced Decohesion (HEDE) and the Hydrogen-Enhanced Localized Plasticity (HELP) mechanism to the HELP-dominated mechanism. Collectively, these findings demonstrate that Cu alloying significantly enhances the resistance of austenitic low-density steels to hydrogen embrittlement.

## 1. Introduction

Fe-Mn-Al-C system low-density steels have emerged as a promising new-generation automotive steel grade, owing to their excellent overall performance and remarkable weight reduction efficacy [1,2]. Nevertheless, like other high-strength steels, they are plagued by the issue of hydrogen embrittlement [3,4]. Hydrogen embrittlement manifests as an unpredictable and abrupt fracture failure, which not only poses a severe threat to the safety of vehicles and passengers but also incurs substantial economic and reputational losses for automobile manufacturers [5,6,7,8,9]. Currently, mitigating hydrogen embrittlement constitutes one of the key technologies and fundamental pillars in the application technology framework of high-strength steels [10,11,12,13]. It has evolved into a critical scientific bottleneck restricting the development and application of high-strength Fe-Mn-Al-C system low-density steels [5,14,15,16], and enhancing their resistance to hydrogen embrittlement has thus become an imperative task in the research and development of high-strength or ultra-high-strength low-density steels [5,17,18,19].

The higher the strength level of high-strength steels, the greater their susceptibility to hydrogen embrittlement and the more severe the resultant hydrogen damage [20,21,22]. Wang et al. [23] reported that the critical hydrogen concentration for hydrogen-induced delayed cracking in AISI 4135 steel with a strength of 1320 MPa is 0.20 wt.ppm; when the steel strength increases to 1450 MPa, the critical hydrogen concentration decreases to approximately 0.06 wt.ppm, signifying a marked rise in hydrogen embrittlement susceptibility. Most high-strength Fe-Mn-Al-C automotive steels are high-manganese steels. Owing to the ability of Mn to expand the austenite phase field, their microstructure is predominantly austenitic matrix with a small amount of ferrite. Kim et al. [24] investigated the hydrogen embrittlement resistance of high-strength steels with different microstructures, revealing that austenite exhibits high hydrogen solubility and a low hydrogen diffusion coefficient. The hydrogen embrittlement susceptibility of austenite and pearlite is lower than that of martensite, with martensitic steels being more prone to hydrogen embrittlement. The research team led by Kim NJ from POSTECH, South Korea studied the microstructure and mechanical properties of Fe-Mn-Al-C low-density steels, achieving a tensile strength exceeding 1600 MPa [25]. Given the high strength of Fe-Mn-Al-C steels, hydrogen embrittlement becomes almost inevitable. Therefore, strengthening research on hydrogen damage in Fe-Mn-Al-C steels is of great significance for their industrial production and application.

Studies have demonstrated that Cu alloying in TWIP steels enhances their hydrogen embrittlement resistance by forming a Cu-rich oxide layer, which inhibits hydrogen diffusion into the steel matrix [26,27]. However, no studies have yet reported whether Cu addition can improve the hydrogen embrittlement resistance of austenitic low-density steels. Therefore, this work investigates the effects of Cu alloying and annealing processes on the hydrogen embrittlement fracture resistance of Fe-28Mn-10Al-1C (wt%) austenitic low-density steel. Hydrogen trapping behavior was characterized via thermal desorption spectroscopy (TDS) experiments, and the activation energy of hydrogen traps was calculated to identify trap types. Slow strain rate tensile (SSRT) tests were conducted to assess hydrogen embrittlement susceptibility based on elongation loss. Fracture surface morphology analysis was performed to explore the fracture mechanism, and the hydrogen embrittlement behavior of Cu-containing austenitic low-density steel was elucidated in conjunction with hydrogen embrittlement mechanisms.

## 2. Materials and Methods

### 2.1. Materials

The Fe-28Mn-10Al-1C-(0,3) Cu steels were fabricated at the Zhuozhou Base of the China Iron and Steel Research Institute Group. Initial melting of raw materials was conducted in a 50 kg vacuum medium-frequency induction furnace, followed by casting into ingots using cast iron molds and slow cooling to ambient temperature. The ingots were then homogenized at 1050 °C for 2 h, hot-forged starting at 950 °C, air-cooled post-forging, and sectioned into small billets. These billets underwent reheating at 1000 °C for 1 h prior to hot rolling, which comprised 10 passes with an approximate 20% reduction per pass, achieving an overall reduction of 87.5%. The resultant hot-rolled sheets were subjected to a solution treatment at 950 °C for 1–2 h, followed by water quenching, before cold rolling. Cold rolling was performed in 10 passes with a cumulative reduction of 60%. Chemical compositions, determined via inductively coupled plasma (ICP) analysis of samples extracted from the forged billets, are presented in Table 1. The cold-rolled Fe-28Mn-10Al-1C-(0,3) Cu (wt%) steels were designated as LZ-0Cu (Cu-free) and LZ-3Cu (3 wt% Cu), respectively. Subsequently, dried specimens were annealed in a box-type resistance furnace at 1000 °C for 0.5 h and quenched in water, yielding the final TH-0Cu and TH-3Cu samples.

### 2.2. Electrochemical Hydrogen Charging and TDS

**Figure 1:**
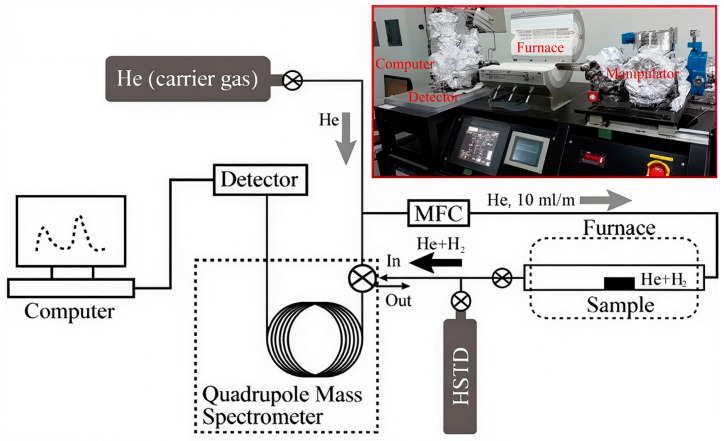
Thermal Desorption Spectroscopy (TDS) device.

Hydrogen-charged specimens were prepared by wire-cutting the experimental steel into 15 mm × 30 mm rectangular coupons and dog-bone-shaped tensile specimens. These were sequentially abraded using SiC papers from 320# to 2000#, mechanically polished, ultrasonically cleaned in ethanol, and dried under a nitrogen stream. Electrochemical hydrogen charging was conducted at ambient temperature using a platinum counter electrode and a saturated calomel reference electrode (SCE). The electrolyte comprised a 0.1 mol/L NaOH aqueous solution with 1 g/L thiourea additive to retard hydrogen effusion during storage. Charging was performed at a current density of 10 mA/cm^2^ for 24 h. Post-charging, specimens were rinsed with deionized water and ethanol, dried, and immediately sealed in a vacuum desiccator. To minimize hydrogen loss, thermal desorption spectroscopy (TDS) measurements were initiated within 5 min of completing the charging process. Quantitative analysis of desorbed hydrogen was carried out using a JTF-20A TDS system (JianTong Technology, Dezhou, China) under a high-purity argon atmosphere (99.999%). Specimens were heated from room temperature to 700 °C at constant heating rates of 100 °C/h, 200 °C/h, and 400 °C/h. Each TDS run was repeated three times to ensure reproducibility. A schematic of the experimental setup is presented in Figure 1.

### 2.3. The Slow Strain Rate Test (SSRT)

The surfaces of electrochemically hydrogen-charged tensile specimens were galvanized to prevent hydrogen outgassing. Uniaxial tensile tests were then conducted on both as-received and hydrogen-charged specimens using an electronic universal testing machine at a constant strain rate of 1 × 10^−4^ s^−1^. Each test was repeated three times to ensure statistical reliability. To ensure statistical rigor, the average value obtained from three repeated experiments was adopted, and the difference between the experimental data and the average value was set as the confidence interval. The hydrogen embrittlement susceptibility index (*HEI*) was calculated using the relative reduction in elongation (δ) before and after hydrogen charging, as defined by Equation (1) [28]:

(1) $$HEI(\%)=(1-\frac{TEL_{H}}{TEL_{0}})\times 100\%$$

where *HEI* denotes the hydrogen embrittlement susceptibility index, *TEL*_0_ and *TEL*_H_ represent the total elongation at fracture of the as-received and hydrogen-charged specimens, respectively. A higher *HEI* value signifies increased susceptibility to hydrogen-assisted cracking.

### 2.4. Microstructural Characterization

Austenite grain and boundary characteristics were characterized via electron backscatter diffraction (EBSD) using a high-resolution scanning electron microscope (HRSEM; S8000, TESCAN, Brno, Czech Republic) equipped with an Oxford Instruments EBSD detector. Data acquisition and analysis were performed using Aztec Crystal software v.2.1. Specimens for EBSD analysis were polished with 0.02-μm colloidal silica (OPS) for 15 min to achieve an atomically smooth surface. Microstructural constituents and fracture surface morphologies were examined using a field-emission scanning electron microscope (FE-SEM; S-4800, Hitachi, Tokyo, Japan) equipped with energy-dispersive X-ray spectroscopy (EDS; X-Max 80, Oxford Instruments, Abingdon, UK). EDS elemental mapping was conducted at an accelerating voltage of 30 kV with a working distance of 10 mm to ensure optimal spatial resolution.

## 3. Results

### 3.1. Hydrogen Desorption Behavior

**Figure 2:**
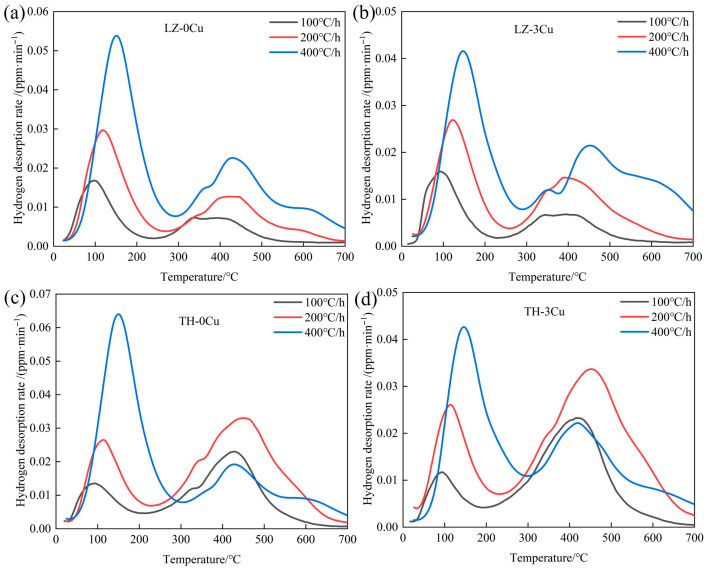
The TDS curves of samples hydrogen-charged at different heating rates: (**a**) LZ-0Cu; (**b**) LZ-3Cu; (**c**) TH-0Cu; (**d**) TH-3Cu.

To investigate the hydrogen desorption behavior of differently processed specimens, TDS was performed from room temperature to 700 °C at constant heating rates of 100, 200, and 400 °C/h. The hydrogen desorption rate profiles are presented in Figure 2. Two prominent desorption peaks emerged around 300 °C: a low-temperature peak (LTP) associated with reversible hydrogen trapping and a high-temperature peak (HTP) attributed to irreversible trapping. Deconvolution analysis revealed that the HTP comprised multiple overlapping sub-peaks, indicating heterogeneous trap sites. According to established conventions, hydrogen released below 300 °C corresponds to weakly bound reversible hydrogen, whereas that released above 300 °C represents strongly trapped irreversible hydrogen. The integrated areas under the LTP and HTP were calibrated to quantify the respective hydrogen contents.

In the low-temperature regime, both cold-rolled and annealed specimens exhibited analogous trends: increasing heating rates induced a linear shift in the LTP to higher temperatures (Figure 2a,b), accompanied by a proportional increase in peak intensity. This behavior is consistent with thermally activated hydrogen detrapping kinetics, where faster heating rates suppress diffusion-driven hydrogen loss prior to desorption. In the high-temperature regime, cold-rolled specimens retained similar peak evolution patterns to the LTP but with diminished intensities, suggesting reduced irreversible trapping capacity. Conversely, annealed specimens displayed distinct HTP characteristics: at 100 and 200 °C/h, the HTP intensity and area exceeded those of the LTP (Figure 2c,d), indicating a predominance of irreversible trapping sites. Notably, increasing the heating rate to 400 °C/h resulted in a 32% reduction in HTP area for annealed specimens, attributed to accelerated thermal detrapping outpacing trap site occupation during heating. These results underscore the critical influence of heating rate on capturing metastable high-energy trap populations in annealed microstructures.

Reversible hydrogen is recognized as the primary driver of hydrogen embrittlement in metallic materials, with higher concentrations of reversible hydrogen in steel exacerbating fracture susceptibility. Gaussian deconvolution was employed to quantify hydrogen contents corresponding to distinct trapping peaks at a heating rate of 100 °C/h, with results tabulated in Table 2. The reversible hydrogen content in LZ-0Cu specimens was determined to be 1.2 wt.ppm, which decreased to 1.1 wt.ppm following Cu alloying. A consistent trend was observed in annealed specimens: TH-0Cu exhibited 1.3 wt.ppm of reversible hydrogen, whereas TH-3Cu showed a reduction to 1.1 wt.ppm. Given that hydrogen embrittlement severity scales with reversible hydrogen content, these findings indicate that Cu alloying reduces the hydrogen embrittlement susceptibility of Fe-28Mn-10Al-1C austenitic low-density steel. The HTP corresponds to hydrogen desorption from irreversible traps, typically associated with second-phase precipitates. While the austenitic matrix of the experimental steel theoretically does not support HTP formation, short-range diffusion of C and Al during TDS heating may induce nucleation of metastable κ-carbide clusters. Despite challenges in morphological characterization due to their nanoscale dimensions, these clusters act as effective irreversible traps, accounting for the HTP observed in cold-rolled specimens.

Koyama et al. [29] confirmed κ-carbide precipitation in Fe-26Mn-11Al-1.2C steel during TDS analysis, with significant growth occurring within 2 min above 400 °C. Variations in lattice misfit between κ-carbides and the austenitic matrix generate spatially varying elastic strain fields, which modulate hydrogen trapping energy and manifest as shifts in desorption peak positions. Our prior work demonstrated that Cu-rich precipitates nucleate at grain boundaries in 3Cu steel after 1 h at 500 °C, with co-precipitation of Cu-rich phases and κ-carbides observed following 3 h of aging at 550 °C [30]. During TDS heating, annealed specimens undergo a “pseudo-aging” process, facilitating the formation of Cu-rich particles. The HTP in annealed 3Cu steel is thus attributed to hydrogen desorption from these Cu-rich precipitates. At the 400 °C/h heating rate, insufficient time for Cu diffusion limits particle coarsening, resulting in fewer effective trapping sites and a 13% reduction in HTP area compared to slower rates (100 and 200 °C/h). This kinetic effect highlights the critical role of heating rate in capturing the metastable equilibrium state of hydrogen trapping in annealed microstructures.

**Figure 3:**
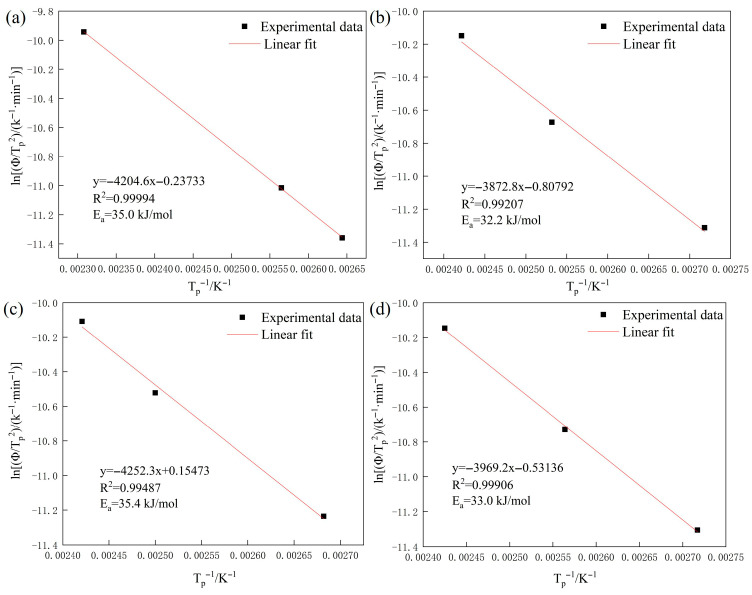
Fitting curve of activation energy for reversible hydrogen traps: (**a**) LZ-0Cu; (**b**) LZ-3Cu; (**c**) TH-0Cu; (**d**) TH-3Cu.

The Kissinger equation predicts a linear correlation between the variables. Linear regression analysis was performed on the peak temperatures obtained at different heating rates, with the resulting fitting curve shown in Figure 3. The activation energy of hydrogen traps can be calculated from the slope of this linear fit. The Kissinger equation is given by Equation (2):

(2) $$\frac{\partial \ln\left(\Phi /T_{p}^{2}\right)}{\partial \left(1/T_{p}\right)}=-\frac{E_{a}}{R}$$

The activation energies (*E*_a_) of reversible hydrogen traps were determined from the Kissinger plots as 35.0 kJ/mol, 32.2 kJ/mol, 35.4 kJ/mol, and 33.0 kJ/mol for LZ-0Cu, LZ-3Cu, TH-0Cu, and TH-3Cu, respectively. The calculation results show that the *E*_a_ of all samples is similar, indicating that the nature of reversible trapping sites remains consistent across all microstructures. These *E*_a_ values align with those reported for hydrogen trapping in microvoids, suggesting that vacancy-aggregated cavities, particularly those formed at grain boundaries during room-temperature charging, serve as the primary reversible traps [31]. Quantitative analysis of TDS peak areas (Table 2) showed that reversible hydrogen accounted for 52.17% and 52.38% of the total hydrogen content in LZ-0Cu and LZ-3Cu, respectively, indicating a predominance of reversible trapping in cold-rolled specimens. In contrast, annealed specimens TH-0Cu and TH-3Cu exhibited significantly lower fractions of reversible hydrogen (43.33% and 30.56%, respectively), suggesting a shift toward irreversible trapping mechanisms. This transition is attributed to the formation of high-energy trapping sites, such as κ-carbides and Cu-rich precipitates, during annealing. The reduced fraction of diffusible hydrogen in annealed specimens correlates with their enhanced resistance to hydrogen embrittlement, as irreversible traps effectively sequester hydrogen in immobile states, mitigating the accumulation of diffusible hydrogen at potential crack initiation sites.

### 3.2. Hydrogen Embrittlement Susceptibility

**Figure 4:**
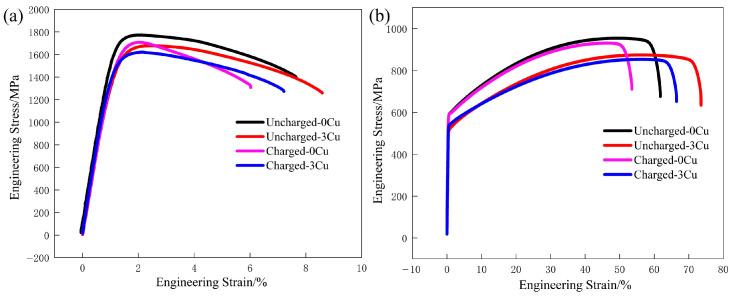
Engineering stress–strain curves of the investigated Fe-28Mn-10Al-1C-(0,3) Cu steel with and without charging: (**a**) cold-rolled state; (**b**) annealed state.

Slow strain rate tensile (SSRT) tests were performed on both uncharged and 24 h pre-charged specimens to quantify their hydrogen embrittlement susceptibility. Engineering stress–strain curves for these specimens are presented in Figure 4, with hydrogen embrittlement indices (*HEI*) calculated via Equation (1) and summarized in Table 3. Hydrogen charging induced consistent reductions in both ultimate tensile strength (UTS) and elongation across all specimens. For cold-rolled LZ-0Cu, the UTS decreased from 1772 MPa to 1708 MPa (−3.6%), accompanied by a drop in uniform elongation (UE) from 7.65% to 6.02% (−21.31%), yielding an *HEI* of 21.31%. In contrast, LZ-3Cu exhibited a less severe degradation: UTS declined from 1677 MPa to 1621 MPa (−3.3%), with UE decreasing from 8.95% to 7.21% (−19.4%) and a corresponding *HEI* of 16.07%. Annealed specimens displayed more pronounced elongation losses upon charging, yet with lower *HEI* values. TH-0Cu showed a reduction in total elongation from 61.90% to 53.51% (−13.5%), while TH-3Cu exhibited a decrease from 73.50% to 66.48% (−9.6%), resulting in *HEI* values of 13.5% and 9.6%, respectively. The reduction in plasticity loss (with a 5.24% decrease in *HEI*) and the corresponding increase in post-hydrogen-charging elongation (7.61% vs. 6.02%) of the LZ-3Cu steel relative to the LZ-0Cu steel reflect the role of copper in suppressing hydrogen-induced plastic degradation. Similarly, the data obtained after annealing further confirm this conclusion. These findings demonstrate a clear trend: annealing treatment combined with Cu addition enhances both ductility and resistance to hydrogen embrittlement, as evidenced by the reduced *HEI* values and mitigated property degradation.

**Figure 5:**
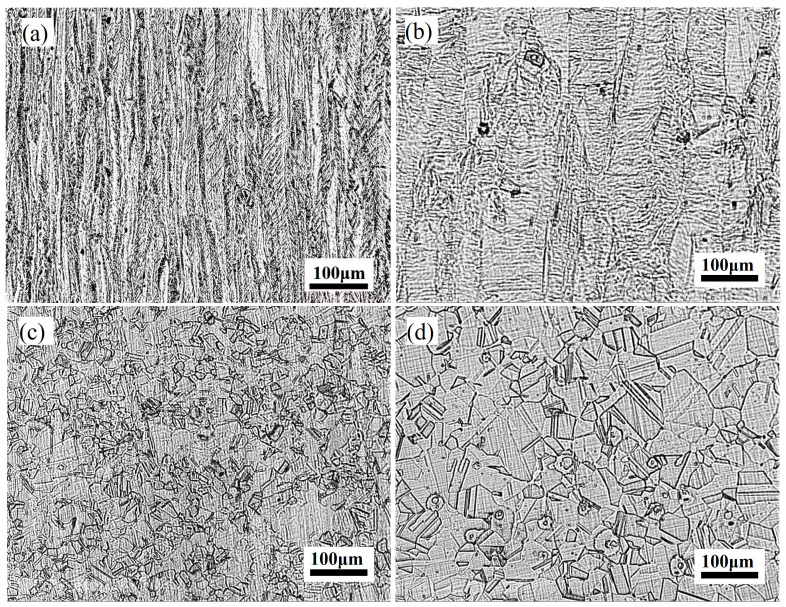
Microstructure of the investigated Fe-28Mn-10Al-1C-(0,3) Cu steel: (**a**) LZ-0Cu; (**b**) LZ-3Cu; (**c**) TH-0Cu; (**d**) TH-3Cu.

The microstructural evolution of the specimens is depicted in Figure 5. Following cold rolling, grains exhibit pronounced elongation along the rolling direction, adopting a lamellar morphology interspersed with mechanical twins. Severe plastic deformation during cold rolling induces extensive dislocation multiplication and the formation of tangled networks, resulting in significant work hardening. This phenomenon restricts dislocation mobility, thereby enhancing yield strength. Concurrently, the increased dislocation density and tangled structure reduce the material’s capacity for uniform deformation, manifesting as diminished elongation. Consequently, cold-rolled specimens are characterized by high strength and low ductility.

Annealing treatment promotes significant grain coarsening and reduces dislocation density, thereby weakening dislocation-mediated strengthening mechanisms and decreasing yield strength. Simultaneously, the formation of recrystallized equiaxed austenite grains homogenizes the microstructure, facilitating dislocation slip and cross-slip across larger distances. This results in enhanced uniform deformation capacity and a substantial increase in total elongation. Cu alloying further exacerbates grain coarsening, as evidenced by the larger grain sizes observed in TH-3Cu compared to TH-0Cu. According to the Hall-Petch relationship, the reduced grain boundary density associated with coarser grains weakens resistance to dislocation motion, thereby contributing to the observed reduction in strength. Concurrently, the larger grain size promotes more homogeneous plastic deformation and mitigates local stress concentrations, which accounts for the enhanced elongation observed in TH-3Cu. These results underscore the critical role of microstructural homogenization and grain coarsening in improving the hydrogen embrittlement resistance of annealed, Cu-alloyed specimens.

Tensile test results confirm that hydrogen charging induces consistent reductions in both ultimate tensile strength and elongation across all specimens, with annealed variants exhibiting lower *HEI* and thus diminished susceptibility. Specifically, the *HEI* of TH-0Cu decreased from 21.3% to 13.5%, while TH-3Cu displayed the lowest *HEI* of 9.6%. According to the established threshold in hydrogen embrittlement research [32], materials with *HEI* < 25% are considered resistant to environmental hydrogen-assisted fracture, indicating that TH-3Cu possesses excellent hydrogen embrittlement resistance.

Analysis shows that the austenite grains increase after annealing treatment. Because austenite has a relatively low hydrogen diffusion rate, it can effectively slow down the process of hydrogen diffusion into the interior of steel. Previous studies have shown that the accumulation of hydrogen in local areas of materials can cause significant internal stress, which in turn leads to stress concentration, increases the sensitivity of steel to hydrogen embrittlement, and ultimately may result in material fracture [33]. Annealing twins formed during the annealing process help refine the grain structure, and grain refinement can effectively alleviate the stress concentration at grain boundaries, thereby reducing hydrogen embrittlement sensitivity. In addition, the addition of Cu elements can enhance the coordinated deformation capacity of the material structure, making the grain deformation near the hydrogen accumulation area more coordinated, thereby alleviating the risk of fracture caused by local stress concentration. In conclusion, both annealing treatment and copper alloying can effectively reduce the hydrogen embrittlement sensitivity of light steel.

## 4. Discussion

### 4.1. Influence of Annealing Treatment and Cu Addition on Hydrogen Content Variation in Steel

**Figure 6:**
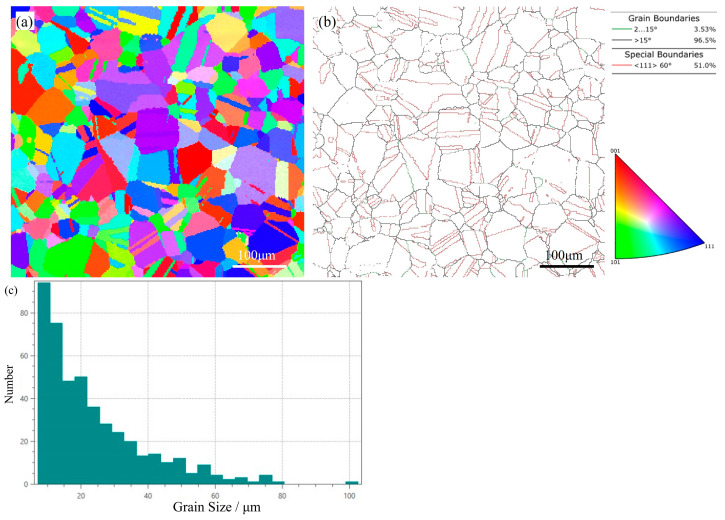
The EBSD maps of the annealed Fe-28Mn-10Al-1C-3Cu steel: (**a**) inverse pole figure (IPF); (**b**) boundary map; (**c**) the grain size distribution map.

As indicated in Table 2, the reversible hydrogen content in cold-rolled steel is marginally lower than that in annealed steel. Based on the characteristics of reversible hydrogen trapping sites, such variations in reversible hydrogen content are primarily attributed to the evolution of grain boundary structures during annealing. The EBSD map of TH-3Cu (Figure 6) reveals that annealing-induced recrystallization generates high-angle grain boundaries (HAGBs, >15°, marked by black solid lines). These HAGBs, characterized by larger free volumes and localized strain fields, exhibit enhanced hydrogen adsorption capacity. Additionally, the formation of annealing twins (e.g., Σ3 twin boundaries, highlighted by red solid lines) increases the grain boundary density, thereby providing additional reversible hydrogen trapping sites. Notably, despite the increased total diffusible hydrogen content in annealed steel, hydrogen atoms are distributed more homogeneously. A higher reversible hydrogen content does not equate to elevated local hydrogen concentrations at embrittlement-susceptible interfaces. For instance, a semi-quantitative analysis using the grain boundary area method—leveraging the inverse relationship between grain boundary area and average grain size—demonstrates that the desorbed hydrogen per unit grain boundary area in annealed steel is lower than that in cold-rolled steel, indicating a reduced hydrogen concentration at grain boundaries.

**Figure 7:**
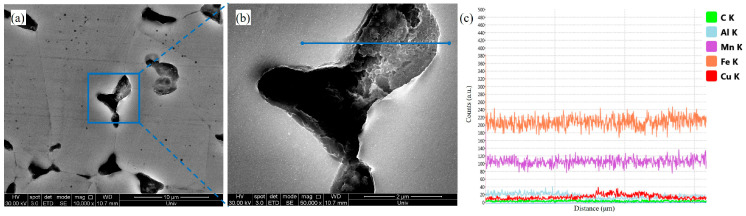
The SEM micrographs of the annealed Fe-28Mn-10Al-1C-3Cu steel after TDS: (**a**) the SEM image; (**b**) local magnification image from (**a**); (**c**) the energy dispersive spectroscopy line scan image.

Although Cu exhibits substantial solubility in austenite, localized segregation during annealing forms Cu-rich clusters, a phenomenon attributed to the combined effects of high cumulative cold rolling reduction and subsequent heat treatment. Severe plastic deformation introduces dense dislocation networks, twin intersections, and twin-defect interfaces, which serve as preferential sites for Cu segregation. During annealing, rapid short-range diffusion of Cu along dislocations facilitates the formation of nanoscale Cu-rich clusters and precipitates at grain boundaries [34], as confirmed by EDS line scans (Figure 7). These Cu precipitates and dispersed clusters homogenize the microstructure, mitigate local defect accumulation, and promote austenite grain growth, thereby reducing the density of high-energy interfaces. Consequently, the formation of triple junctions—susceptible to hydrogen accumulation and crack initiation—is suppressed. This mechanism is identified as the primary pathway by which Cu alloying enhances the resistance to hydrogen embrittlement in low-density steels. The exceptional performance of TH-3Cu is primarily attributed to this transition from localized hydrogen enrichment at stress-concentrated interfaces to a more uniform distribution within the matrix.

Samusawa et al. [35] reported that a Cu-enriched layer forms on the surface of Cu-containing steels, altering the mechanism of the hydrogen evolution reaction (HER). Specifically, surface-accumulated Cu catalyzes the recombination of adsorbed hydrogen atoms into molecular H_2_, thereby suppressing hydrogen absorption into the substrate. This dual function of promoting HER recombination and impeding hydrogen permeation explains the reduced hydrogen desorption in TH-3Cu compared to TH-0Cu, though the limited reduction suggests a discontinuous or porous nature of the protective layer. Second-phase precipitates act as irreversible hydrogen traps, simultaneously sequestering non-diffusible hydrogen and accelerating hydrogen diffusion in adjacent regions via a trapping-induced concentration gradient. The elevated total desorbed hydrogen content in TH-3Cu is thus attributed to the attractive trapping effect of Cu precipitates/Cu-rich clusters. Hi et al. [36] similarly ascribed the enhanced resistance to hydrogen-induced cracking in Cu-bearing pipeline steels to nanoscale Cu-rich precipitates, which serve as high-affinity trapping sites. Lin et al. [13] further demonstrated that Cu precipitates improve hydrogen trapping efficiency in martensitic steels by introducing additional trapping sites.

While nanoscale carbides have been extensively studied as irreversible hydrogen traps in steels—with direct evidence provided by deuterium-assisted 3D atom probe tomography—Cu precipitates/Cu-rich clusters are hypothesized to exhibit analogous behavior. As shown in Figure 7, Cu-rich B2 particles preferentially nucleate at pre-existing hydrogen trapping sites (e.g., grain boundaries and phase interfaces), introducing misfit dislocations that transform reversible traps into irreversible ones. These stable traps immobilize hydrogen atoms, impede their diffusion and segregation to stress-concentrated regions, refine the grain structure, and pin mobile dislocations—collectively mitigating hydrogen embrittlement susceptibility.

### 4.2. Hydrogen Embrittlement Mechanism Analysis

**Figure 8:**
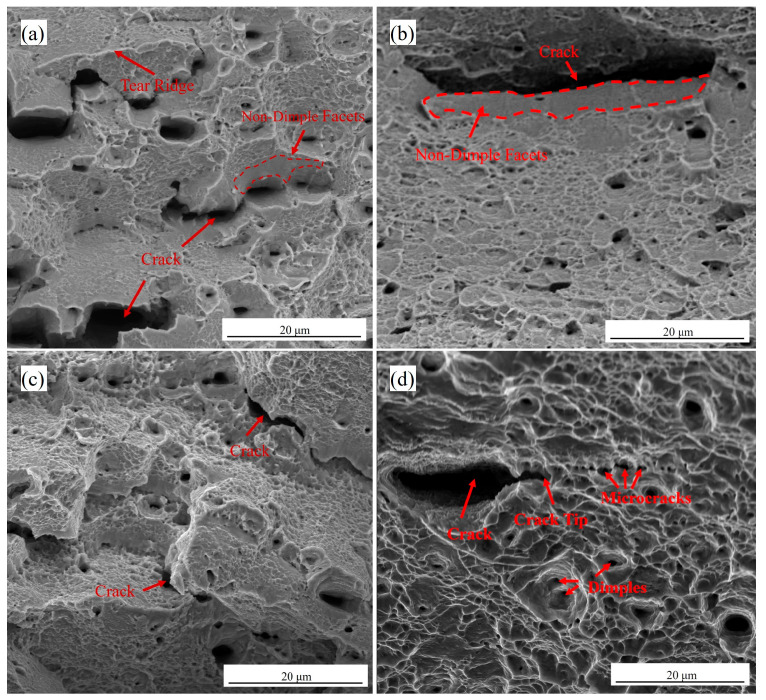
High-magnification SEM images of the fracture surfaces after hydrogen charging: (**a**) LZ-0Cu; (**b**) LZ-3Cu; (**c**) TH-0Cu; (**d**) TH-3Cu.

Figure 8 presents SEM micrographs of the tensile fracture surfaces for the four hydrogen-charged specimens. The LZ-0Cu fracture morphology exhibits tear ridges, micro-cleavage facets, sparse dimples, and visible crack initiation, characteristic of quasi-cleavage fracture. LZ-3Cu displays fine dimples interspersed with quasi-cleavage platforms and distinct secondary cracks, indicative of quasi-cleavage fracture without severe embrittlement. TH-0Cu features intergranular facets with cracks propagating along triple junctions, accompanied by isolated dimples and cleavage-like regions, indicating a mixed-mode brittle-ductile fracture. In contrast, TH-3Cu exhibits uniformly distributed dimples of varying sizes, a hallmark of microvoid coalescence and ductile fracture. These observations suggest that Cu alloying promotes a transition from mixed-mode to fully ductile fracture behavior. Collectively, the fracture morphologies correlate strongly with the mechanical property data, confirming that Cu addition enhances the resistance of Fe-28Mn-10Al-1C steel to hydrogen-assisted cracking.

The Hydrogen-Enhanced Localized Plasticity (HELP) mechanism posits that hydrogen facilitates dislocation motion, leading to localized plastic deformation, strain concentration, and microcrack initiation [37]. This theory effectively explains the hydrogen embrittlement behavior observed in 3Cu steel. Specifically, hydrogen atoms segregate to dislocation cores, reducing the Peierls stress and lowering the critical resolved shear stress required for dislocation slip. Concurrently, hydrogen accelerates dislocation multiplication, resulting in elevated local dislocation densities. Under the influence of hydrogen, dislocation activity becomes confined to narrow regions (e.g., crack tips), forming highly localized plastic zones. The resultant high local strain promotes microvoid nucleation, while hydrogen segregation at crack tips generates hydrostatic pressure that drives crack propagation (Figure 8d). The main crack propagates by coalescing with microcracks and voids at the tip, ultimately leading to plastic instability and ductile tearing.

After hydrogen charging, TH-0Cu exhibits intergranular fracture morphology with distinct intergranular cracks, accompanied by scattered dimples—characterizing a brittle-dominated mixed fracture mode with localized plasticity, which is typical of hydrogen-induced failure in high-strength steels. The fracture mechanism involves triple junction cracking and microvoid-induced intergranular cracking arising from slip localization along grain boundaries; progressive crack propagation ultimately results in catastrophic failure (Figure 8c). This behavior stems from hydrogen adsorption at grain boundaries, which diminishes grain boundary cohesive strength via the Hydrogen-Enhanced Decohesion (HEDE) mechanism [38,39]. During deformation, hydrogen desorbs from reversible trapping sites (e.g., dislocations and vacancies) and accumulates at grain boundaries, forming continuous “hydrogen channels.” Concurrently, hydrogen enrichment at triple junctions—where elastic mismatch and stress concentration are maximized—facilitates crack initiation at the early deformation stage. Localized plastic deformation preferentially occurs around triple junctions during loading. The synergistic effect of deformation-induced high stress and hydrogen enrichment accelerates dislocation motion, triggering localized plasticity (consistent with HELP theory) and leading to limited dimple formation. The coexistence of intergranular facets and localized dimples on the fracture surface thus reflects the coupled action of HEDE and HELP mechanisms.

Based on the aforementioned fracture mechanism analysis, unpinning of dislocations enables their movement, during which they transport hydrogen atoms and facilitate hydrogen accumulation at defect sites (predominantly grain boundaries). Collectively, these observations indicate that Cu-rich precipitates function as irreversible hydrogen traps, effectively constraining hydrogen diffusion and aggregation. Additionally, they exert a dual role in pinning dislocations and refining grain structures, thereby impeding hydrogen mobility. In low-density austenitic steels, trace additions of Cu induce the precipitation of finely dispersed second-phase particles. These particles act as irreversible hydrogen traps to sequester hydrogen atoms, refine the microstructure, and pin mobile dislocations—synergistically enhancing resistance to hydrogen embrittlement.

## 5. Conclusions

This study investigates the hydrogen embrittlement behavior of Fe-28Mn-10Al-1C-(0,3) Cu austenitic low-density steels after hydrogen charging under cold-rolled (LZ) and annealed (TH) conditions. The main research conclusions are as follows:

1. Annealing treatment and Cu addition have little effect on the activation energy of reversible hydrogen traps; based on the activation energy, the types of reversible hydrogen traps are determined to be the same. Compared with 0Cu steels, the reversible hydrogen contents in LZ-3Cu and TH-3Cu decrease by 0.1 wt.ppm and 0.2 wt.ppm, respectively, indicating that Cu addition can inhibit the increase in reversible hydrogen content to a certain extent.
2. After hydrogen charging, both the UTS and UE of the specimens decrease. The hydrogen embrittlement index (*HEI*) of LZ-0Cu decreases from 21.3% to 16.0% compared with that of LZ-3Cu, and the *HEI* of TH-0Cu decreases from 13.5% to 9.6% compared with that of TH-3Cu. TH-3Cu exhibits the lowest *HEI*. Cu precipitates homogenize the microstructure. This leads to a transition of hydrogen distribution from severe local concentration to a more uniform state. Meanwhile, as stable irreversible hydrogen traps, Cu precipitates can hinder hydrogen diffusion and segregation.
3. Due to Cu alloying, the fracture morphology tends to transform from mixed brittle-ductile fracture to ductile fracture, and the fracture mechanism also changes from the coupled HEDE and HELP mechanism to the HELP mechanism. In conclusion, copper alloying can significantly improve the hydrogen embrittlement resistance of austenitic low-density steels.

## Figures and Tables

**Table 1:** Main chemical composition of the investigated Fe-28Mn-10Al-1C-(0,3) Cu steel.

Alloy	Fe	Mn	Al	C	Cu
0Cu	Bal.	28.15	10.02	0.98	0
3Cu	Bal.	28.38	10.08	1.04	3.01

**Table 2:** Hydrogen content measured by TDS.

Specimens	Reversible Hydrogen Content (wt.ppm)	Irreversible Hydrogen Content (wt.ppm)	Total Hydrogen Content (wt.ppm)
LZ-0Cu	1.2 ± 0.1	1.1 ± 0.2	2.3 ± 0.3
LZ-3Cu	1.1 ± 0.1	1.0 ± 0.1	2.1 ± 0.2
TH-0Cu	1.3 ± 0.2	1.7 ± 0.2	3.0 ± 0.4
TH-3Cu	1.1 ± 0.1	2.5 ± 0.5	3.6 ± 0.6

**Table 3:** Slow strain rate tensile (SSRT) data.

State	Specimens	UTS (MPa)	YS (MPa)	UE (%)	*HEI* (%)
	LZ-0Cu	1772 ± 45	1554 ± 39	7.65 ± 0.41	-
Original state	LZ-3Cu	1677 ± 41	1450 ± 36	8.59 ± 0.52	-
	TH-0Cu	954 ± 22	586 ± 25	61.90 ± 1.83	-
	TH-3Cu	874 ± 25	517 ± 23	73.50 ± 1.77	-
	LZ-0Cu	1708 ± 43	1430 ± 32	6.02 ± 0.63	21.31
After hydrogen charging	LZ-3Cu	1621 ± 37	1336 ± 35	7.21 ± 0.55	16.07
	TH-0Cu	930 ± 31	584 ± 21	53.51 ± 2.11	13.55
	TH-3Cu	853 ± 28	529 ± 27	66.48 ± 1.98	9.55

Note: UTS—ultimate tensile strength, YS—yield strength, UE—uniform elongation.

## Data Availability

The original contributions presented in this study are included in the article. Further inquiries can be directed to the corresponding author.
